# Sodium butyrate-induced autophagy in colorectal cancer unveils the Ca²⁺/CaMKKβ signaling pathway as a potential therapeutic target

**DOI:** 10.1038/s41598-025-29618-7

**Published:** 2025-12-11

**Authors:** Shunli Luo, Ziyin Li, Lianzhi Mao, Suxia Sun

**Affiliations:** 1 Department of Nutrition and Food Hygiene, Guangdong Provincial Key Laboratory of Tropical Disease Research, School of Public Health, Southern Medical University, Guangzhou, 510515 China; 2 Department of Food Hygiene and Nutrition, College of Laboratory Medicine, Hunan University of Medicine, Huaihua, 418000 China

**Keywords:** NaB, Ca^2+^, Colorectal cancer, CaMKKβ, Autophagy, Biochemistry, Cancer, Cell biology, Molecular biology

## Abstract

Colorectal cancer’s autophagy process is facilitated by sodium butyrate (NaB), but the mechanism remains unclear. This study aimed to elucidate the effects and underlying mechanisms of NaB-induced autophagy in colorectal cancer cells. In colorectal cancer cells, NaB has been shown to upregulate the expression of autophagy-related proteins, including microtubule-associated protein 1 A/1B-light chain 3 (LC3), thereby promoting autophagosome formation. Furthermore, NaB induces the activation of Ca^2+^/calmodulin-dependent kinase kinase β (CaMKKβ), AMP-activated protein kinase α (AMPKα), and acetyl-CoA carboxylase (ACC). Moreover, inhibiting CaMKKβ with STO-609 or downregulating CaMKKβ expression using RNA interference significantly attenuated the autophagic effect induced by NaB in colorectal cancer cells, leading to reduced expression of phosphorylated CaMKKβ, AMPKα, and ACC proteins. Furthermore, sequestering cytoplasmic calcium has been found to diminish NaB-induced autophagy and partially inhibit the activation of CaMKKβ, AMPKα, and ACC proteins. The findings indicate that NaB stimulates autophagy in colorectal cancer cells by modulating Ca^2+^/CaMKKβ signaling pathways.

**Supplementary Information:**

The online version contains supplementary material available at 10.1038/s41598-025-29618-7.

## Introduction

Global cancer statistics reveal that colorectal cancer ranks as the third most prevalent malignant tumor and the second leading cause of cancer-related mortality^1^. Currently, the primary modalities for treating colorectal cancer include surgical intervention, radiotherapy, chemotherapy, immunotherapy and endoscopic treatment^2,3^. While the combination of surgery and chemoradiotherapy has been shown to enhance the five-year survival rate of these patients, the occurrence of these side effects remains inevitable. Consequently, there is a pressing need to identify a pharmacological agent that is both environmentally sustainable and safe for the prevention and management of colorectal cancer.

NaB is a sodium salt of a four-carbon short-chain fatty acid, which is primarily derived from the microbial fermentation of soluble dietary fiber in the intestinal tract^4^. Accumulating evidence has indicated that NaB possesses significant anticancer properties, including the ability to induce cell cycle arrest, differentiation, autophagy, and apoptosis in various tumor cell types^5–7^. For instance, in colorectal cancer cells, NaB has been shown to inhibit histone deacetylase (HDAC) activity, thereby regulating autophagy and exerting its anticancer effects^8^. In addition to the aforementioned findings, our research group’s preliminary studies have demonstrated that NaB can inhibit the progression of colorectal cancer by disrupting aerobic glycolysis mediated by the SIRT4/HIF-1α axis^9^. However, despite advances these in understanding, a comprehensive elucidation of the molecular mechanisms by which NaB exerts its anticancer effects remains an ongoing area of research.

The process of autophagy involves the breakdown and recycling of small molecules, such as proteins, organelles, amino acids, and fatty acids, to maintain cell homeostasis. The role of autophagy in tumor cells is highly controversial, as it is widely recognized to function as a double-edged sword^10^. The mainstream view currently holds that in the initial stages of colorectal cancer, autophagy is predominantly regarded as protective, contributing to cellular homeostasis and the prevention of tumorigenesis^11,12^. Conversely, in advanced colorectal cancer, autophagy frequently facilitates tumor survival and metastasis^13,14^. While phagocytosis regulation and the underlying molecular mechanisms have made great progress in recent years, many questions remain.

AMPK is a highly conserved serine/threonine kinase complex that acts as a crucial bioenergetic stress sensor in a wide range of mammalian systems, primarily functioning to enhance cell survival under conditions resembling starvation^15^. Activation of AMPK is facilitated by various upstream kinases, including liver kinase B1 (LKB1), CaMKKβ, and transforming growth factor β-activated kinases. CaMKKβ, positioned upstream of AMPK, also serves as a key downstream effector in Ca^2+^ signaling pathways, with its activity being modulated by changes in intracellular Ca^2+^ levels^16^. Recent studies have shown that the Ca^2+^/CaMKKβ/AMPK signal is closely related to autophagy^17–22^. CaMKK-AMPK signal cascade activation was also observed in Autosomal dominant polycystic kidney cells, which resulted in the inhibition of mTOR signal transduction and induction of autophagy^17^. Our research group has previously demonstrated that NaB can enhance autophagy in colorectal cancer cells via the LKB1/AMPK pathway^23^. Therefore, we are interested in investigating whether NaB can influence the activity of CaMKKβ by modulating cytoplasmic Ca^2+^ levels, leading to the activation of AMPK signaling and subsequent induction of autophagy in colorectal cancer cells.

Based on the aforementioned research findings, we propose the hypothesis that NaB may induce autophagy in colorectal cancer cells by activating the Ca^2+^/CaMKKβ pathway. This hypothesis was tested by using HT29 cells to represent colorectal cancer in vitro. The initial focus of our research was twofold: first, to investigate the effects of NaB on autophagy in colorectal cancer cells, and second, to explore whether NaB-induced autophagy is associated with the Ca²⁺/CaMKKβ signaling cascade.

## Materials and methods

### Reagents and antibodies

Total protein extract was obtained using protease inhibitor and phosphatase inhibitor from Nanjing Kaiji Biotechnology Development Co., LTD. Pancreatic enzyme, fetal bovine serum (FBS), and DMEM medium from Gibco were used for cell culture. Cell Signaling Technology, USA, provided the p-CaMKKβ, ACC, p-ACC, p-AMPKα, AMPKα, GAPDH, and LC3 for this study. RNA extraction was performed using TRIzol and SYBR^®^ Premix Ex TAQTMs II from TAKALA, Japan. Chemiluminescent reagent (ECL) from Pierce Corporation, USA was used for protein detection. Total protein extraction kit from KeyGen Biotech Co., Ltd., Nanjing, China was used for total protein extraction. Lipo 6000TM from Shanghai Biyuntian Biotechnology Co., LTD. Other reagents not specified were purchased from Sigma.

### Cell culture

HT29 colorectal cancer cells were obtained from the American Type Culture Collection (Manassas, VA, USA). These cells were revived using a standard protocol and cultured in DMEM medium supplemented with 10% fetal bovine serum. The cells were passaged every two to three days, and when they reached the logarithmic growth phase, they were digested with 0.25% trypsin and used for experimentation. All cell culture procedures were performed in humidified incubators at 37 °C with 5% CO_2_.

### Western blot

Total protein was extracted from HT29 cells using a protein extraction kit. Following the measurement of total protein, the BCA (bicinchoninic acid) method was used to determine the concentration of total protein. The protein was electrophoretically separated on SDS-PAGE using a total protein sample of 30 g, followed by wet transfer to a PVDF membrane. A solution of 5% skim milk was incubated on the membrane for an hour at room temperature, and then the primary antibody was incubated overnight at 4 °C. We washed the film three times for 10 min each time, incubated it at room temperature with secondary antibody for two hours, washed it three times for 10 min each time, obtained ECL luminescence, and took pictures. Densitometry analysis was performed using ImageJ software (National Institutes of Health, USA).

### Real-time quantitative PCR (RT-PCR)

A total cell RNA extraction was performed on samples collected. In general, RNA samples have an absorbance ratio between 1.8 and 2.0 at 260/280 nm. Subsequently, 450 ng of RNA was reverse-transcribed into cDNA, resulting in a final mixture volume of 25 µL containing SYBR^®^Premix ExTaqTM II, 1 µL of upstream primer, 1 µL of downstream primer, 90 ng of cDNA sample, and DEPC water. Based on the following protocol, 45 cycles of polymerase chain reaction (PCR) were conducted: an initial pre-incubation stage at 95 ℃ for 30 s, followed by a two-step amplification stage at 95 ℃ for 5 s and 60 ℃ for 1 min. This was succeeded by a melting stage at 95 ℃ for 60 s, 30 ℃ for 55 s, and 97 ℃ for 1 s, and a cooling stage at 37 ℃ for 30 s. The PCR products were analyzed using the LightCycler^®^ 96 System. To account for sample variability, GAPDH was used as the internal reference gene. The primer sequences are presented in Table 1.

**Table 1:** Primer sequence.

	Forward sequence	Reverse sequence
LC3	AAGTGGCTGAGTACCGACC	GATCTCCAGCTGCCACAAAC
GAPDH	CAAATTCCATGGCACCGTCA	ATCTCGCTCCTGGAAGATGG

### Transmission electron microscopy

Samples were fixed in a PBS solution with a pH of 7.4 and 2.5% glutaraldehyde for a duration of 30 min following digestion with trypsin, double washing with PBS, and additional fixation with a PBS solution containing a pH of 7.4 and 2.5% glutaraldehyde. Subsequently, the samples underwent further washing with PBS two to three times, fixation with osmic acid, and embedding in Spurr’s Epon before representative areas were sectioned and examined using a transmission electron microscope (Hitachi 7500, Japan).

### Small interfering RNA (siRNA)

Inoculated cells were allowed to grow for about 24 h, and cell density was monitored periodically. As soon as the cell density reached approximately 40%, the Opti-MEM^®^ medium in the six-well plate was replaced with 2 mL. Two aseptic centrifuge tubes were then taken from each dish, with 125 µL of Opti-MEM^®^ Medium added to one tube and 100 pmol of siRNA added to the other tube. The contents of each tube were carefully mixed without vortexing or centrifugation. Additionally, 5 µL of Lipo6000TM transfection reagent was added to the tube containing siRNA, followed by thorough mixing. After allowing the culture medium containing siRNA to equilibrate at room temperature for 5 min, it was combined with the culture medium containing Lipo6000TM transfection reagent and thoroughly mixed. Add to the six-well plate in the corresponding position after 20 min at room temperature. During the 48-hour culture period, the wells were replenished with 2 mL of fresh medium after a 4-hour incubation period in the incubator.

A detailed sequence of siRNA can be found in Table 2.

**Table 2:** The interference sequences of colorectal cancer cells.

Name	Sence	Antisence
Negative control	UUCUCCGAACGUGUCACGUTT	ACGUGACACGUUCGGAGAATT
CaMKKβ siRNA-1	GCCUCUCAUCCUUGAGCAUTT	AUGCUCAAGGAUGAGAGGCTT
CaMKKβ siRNA-2	GGGAAGGCCUUGGAUGUUUTT	AAACAUCCAAGGCCUUCCCTT

### Statistical analysis

All experiments were performed independently at least three times. We analyzed the data using SPSS 25.0 and compared the results using one-way ANOVA. In cases where homogeneity of variance was confirmed, multiple comparisons were conducted using the Least Significant Difference (LSD) method. Alternatively, when variance was found to be non-uniform, corrections were made using the Welch and Brown-Forsythe methods in an approximate F test, with subsequent evaluation of results using Dunnett’s T3. All statistical analyses have *P < 0.05, **P < 0.01, ***P < 0.001 as the level of significance.

## Results

### NaB induces autophagy in colorectal cancer cells

**Fig. 1:**
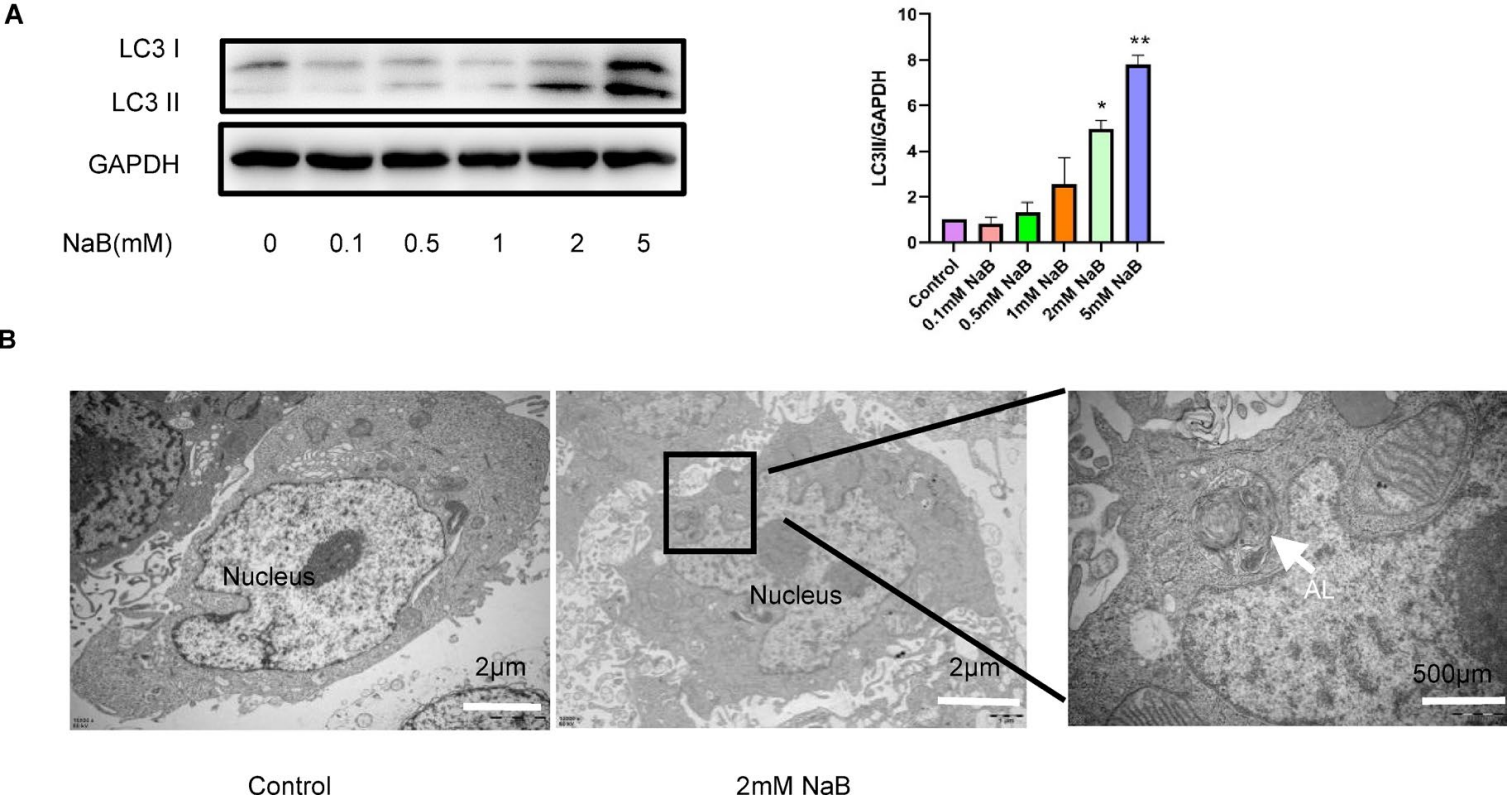
Impact of NaB on autophagy in colorectal cancer cells. (**A**) HT29 cells were incubated in NaB concentrations of 0.1, 0.5, 1, 2, 5 mM for 24 h prior to Western blot analysis. The expression of LC3II protein was examined using Western blotting and densitometry. Data were derived from three independent experiments and are presented as the mean ± standard deviation. (**B**) Autolysosomes were observed in HT29 cells treated with NaB for 24 h using transmission electron microscopy. AL, autolysosome. *P < 0.05.

To investigate the role of NaB in autophagy in colorectal cancer cells, we performed a series of analyses. These included Western blotting to evaluate the expression levels of free LC3-I and lipid-bound LC3II, as well as transmission electron microscopy to observe the formation of autolysosomes. We exposed HT29 colorectal cancer cells to varying concentrations of NaB (0.1, 0.5, 1, 2, and 5 mM) for a duration of 24 h. The findings, illustrated in Fig. 1A, demonstrate that NaB concentrations exceeding 2 mM significantly enhance the expression levels of LC3Ⅱ protein in HT29 cells. Furthermore, a progressive increase in LC3Ⅱ protein expression was observed with escalating NaB concentrations. Transmission electron microscopy analysis revealed the formation of autolysosomes (AL), characterized by a double-layer membrane structure, in HT29 cells treated with 2 mM NaB for 24 h, thereby indicating the induction of autophagy (Fig. 1B). LC3, an essential component of the autophagy pathway, is widely recognized as a biochemical marker for this cellular process^24^. Additionally, Fig. 3B illustrates that NaB resulted in significant increases in LC3 mRNA expression. It appears that NaB induces autophagy in colorectal cells based on the above results.

### NaB activates CaMKKβ, AMPKα and ACC in colorectal cancer cells

**Fig. 2:**
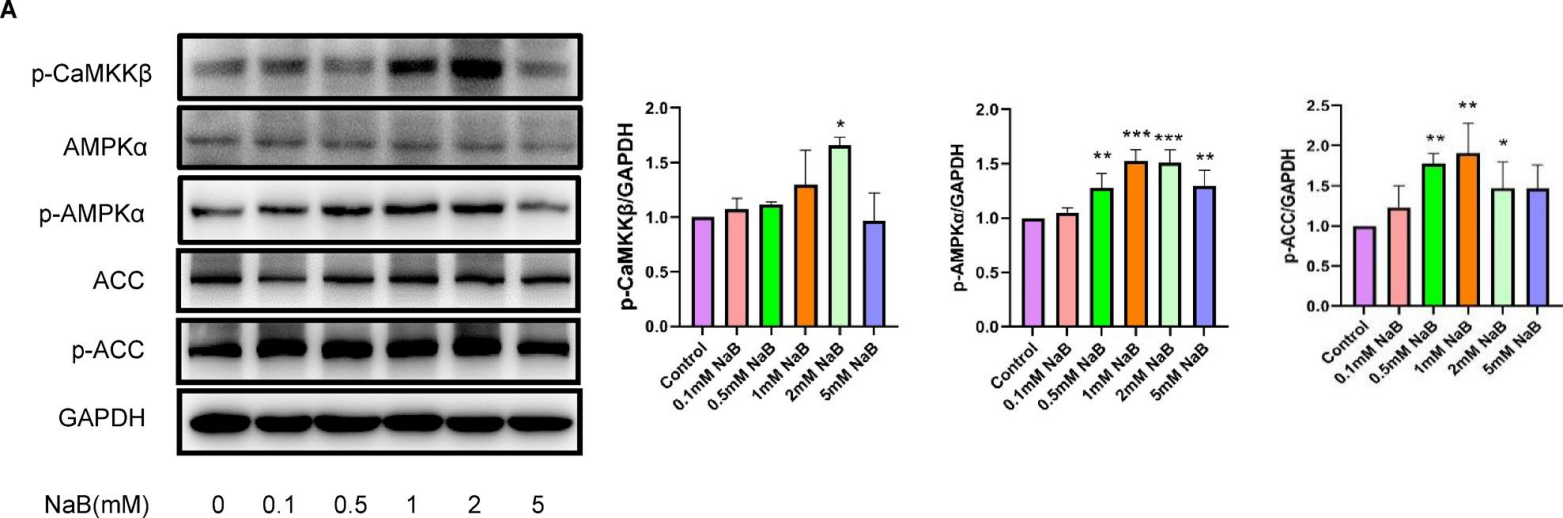
Activation of CaMKKβ, AMPK, and ACC by NaB in colorectal cancer cells. HT29 cells were treated with NaB at concentrations of 0.1, 0.5, 1, 2, and 5 mM for 2 h. Western blotting and densitometry were then used to evaluate the expression levels of AMPKα, p-AMPKα, p-CaMKKβ, ACC, and p-ACC. * *P* < 0.05; **P < 0.01 vs. control.

At present, the CaMKKβ/AMPK signaling pathway is the focus of extensive research, with evidence indicating its involvement in various physiological and pathophysiological processes, such as glucose homeostasis, cancer progression, and the regulation of autophagy^25^. To explore the potential relationship between the autophagic effects of NaB and CaMKKβ/AMPK signaling, western blot analyses were performed on NaB-treated colorectal cancer cells. HT29 cells were treated with varying concentrations of NaB (0.1, 0.5, 1, 2, and 5 mM) for 2 h. As illustrated in Fig. 2, exposure of HT29 cells to 2 mM NaB for 2 h resulted in a significant upregulation of p-CaMKKβ protein expression. Similarly, Fig. 2 demonstrates that treatment with 0.5, 1, 2, and 5 mM NaB for 2 h significantly enhanced the phosphorylation of AMPKα protein. Furthermore, as depicted in Fig. 2, treatment with 0.5, 1, and 2 mM NaB for 2 h significantly increased the expression of p-ACC protein. These findings corroborate the hypothesis that NaB can stimulate the activation of CaMKKβ, AMPKα, and ACC in colorectal cancer cells.

### CaMKKβ signaling is crucial for NaB-induced autophagy in colorectal cancer

**Fig. 3:**
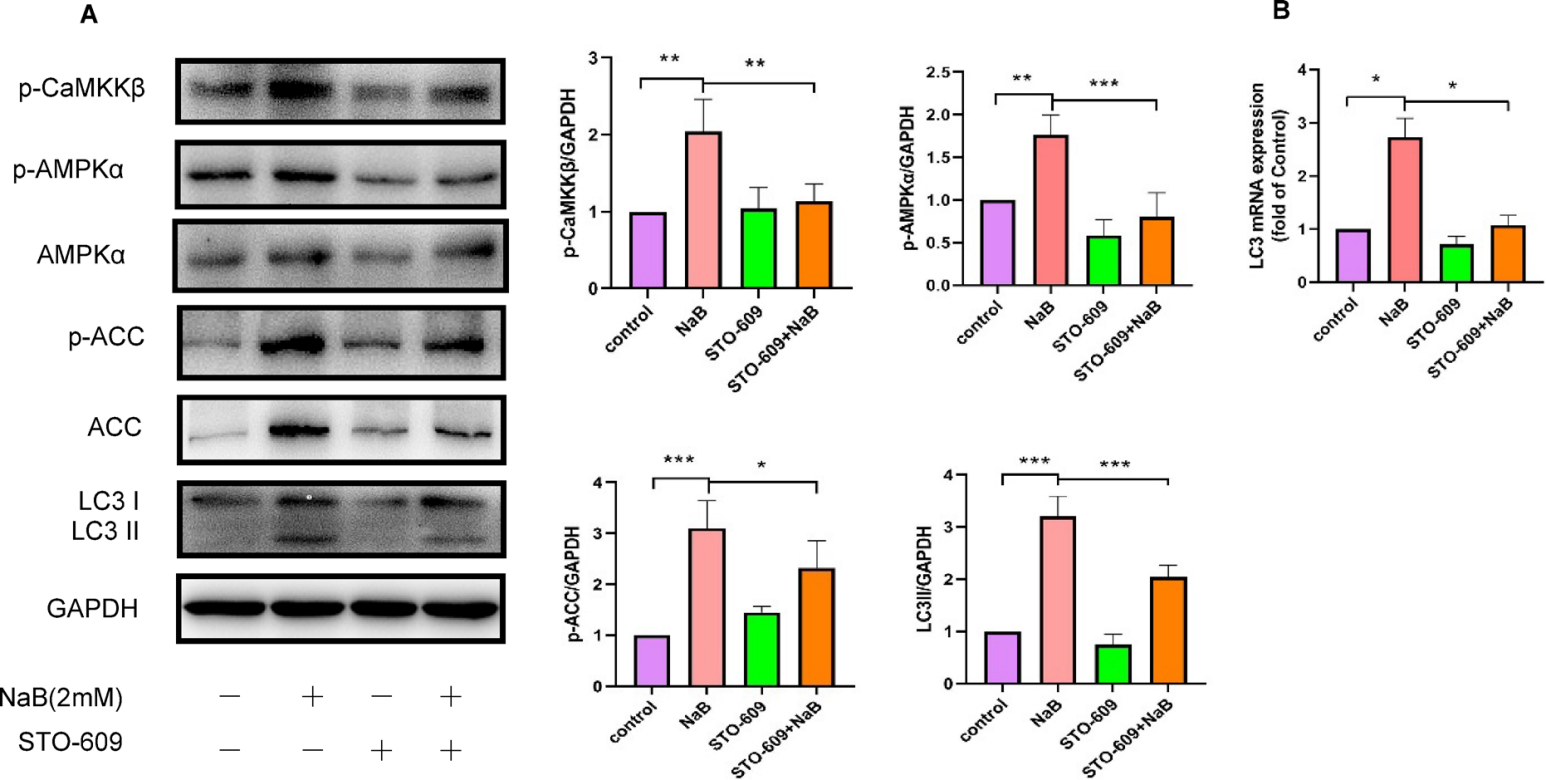
Inhibition of CaMKKβ activity attenuated NaB-induced autophagy in colorectal cancer cells. (**A**) Colorectal cancer cells were pretreated with STO-609 (5 µM) for 30 min, followed by exposure to 2 mM NaB for either 24 h–2 h. Protein lysates were then subjected to Western blotting and band intensities quantified by densitometry; the 24 h treatment group was used to assess LC3-II levels, whereas the 2 h treatment group was employed to examine the expression of AMPKα, ACC, p-CaMKKβ, p-AMPKα, and p-ACC. (B) LC3 mRNA levels in HT29 cells were quantified using quantitative RT-PCR. *P < 0.05, **P < 0.01, ***P < 0.001.

To investigate the potential contribution of CaMKKβ to NaB-induced autophagy in colorectal cancer cells, cells were pretreated with the CaMKKβ-selective inhibitor STO-609 (5 µM) for 30 min prior to exposure to 2 mM NaB. The expression of LC3Ⅱprotein was subsequently assessed via western blot analysis, while the expression of LC3 mRNA was evaluated using fluorescence quantitative PCR. Inhibition of CaMKKβ by STO-609, as expected, significantly blocked phosphorylation of CaMKKβ proteins (refer to Fig. 3A). Furthermore, the findings indicate that the co-administration of STO-609 and NaB resulted in a notable decrease in the NaB-induced phosphorylation of AMPKα and ACC proteins, as demonstrated in Figs. 3A. According to Fig. 3A, the combined treatment of STO-609 and NaB resulted in a significant decreased in the expression level of the LC3II protein compared to the NaB group alone. Similarly, as depicted in Fig. 3B, the expression level of LC3 mRNA was significantly decreased when STO-609 was administered in conjunction with NaB, relative to the administration of NaB alone. These findings indicate that the CaMKKβ signaling pathway is essential for NaB-induced autophagy in colorectal cancer cells.

### CaMKKβ signaling was involved in NaB-induced autophagy in colorectal cancer

To further delineate the contribution of CaMKKβ signaling to NaB-induced autophagy in colorectal cancer cells, CaMKKβ expression was stably silenced in HT29 cells via RNA interference. Thereafter, control and CaMKKβ-silenced cells were exposed to 2 mM NaB to assess the impact of CaMKKβ depletion on autophagic flux.

**Fig. 4:**
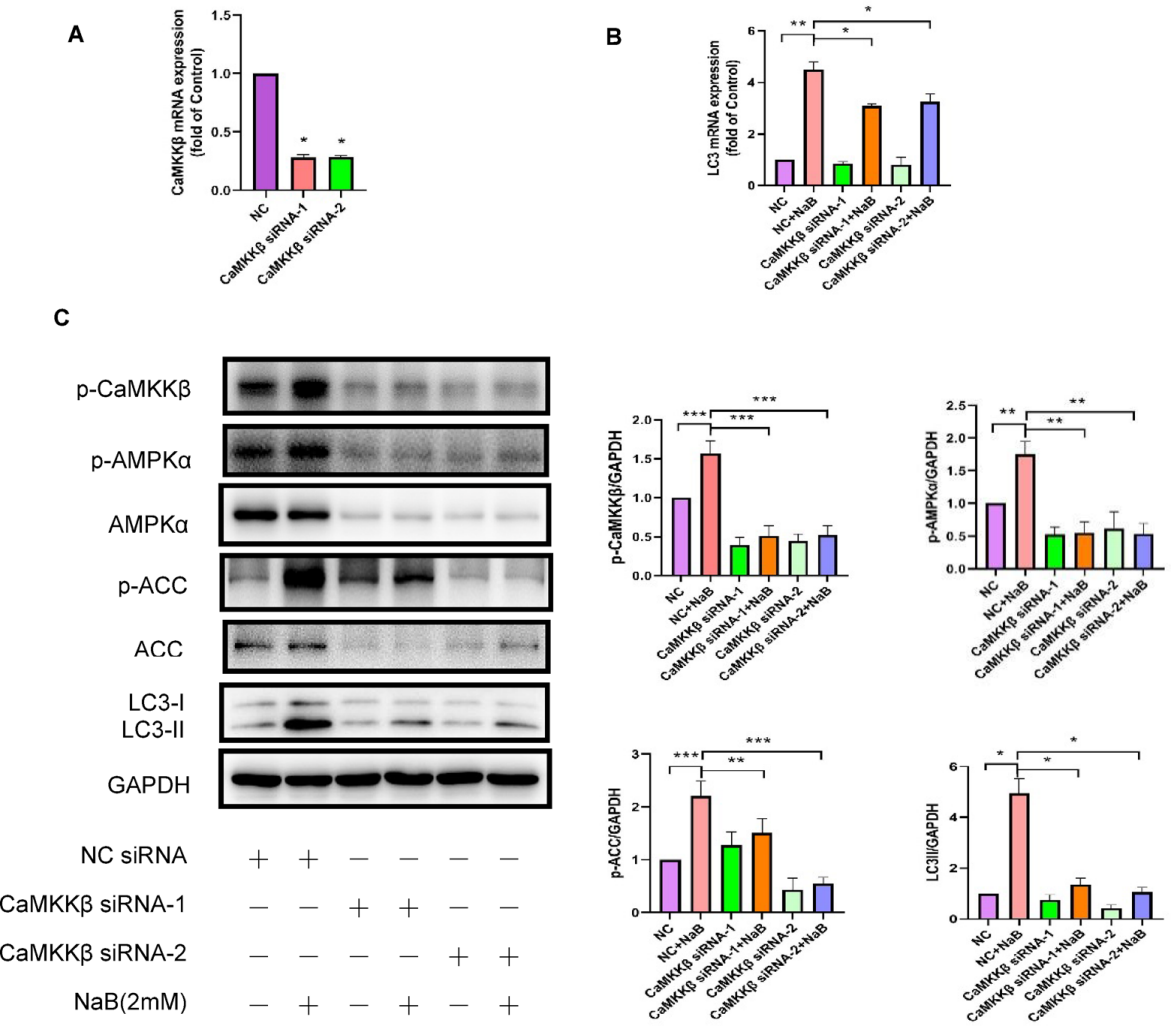
Reducing CaMKKβ expression via RNA interference decreased NaB-mediated autophagy in colorectal cancer cells. (**A**) The HT29 cell line was exposed to either a non-targeting control sequence or one of two specific interference sequences for a duration of 24 h. Thereafter, alterations in CaMKKβ mRNA expression levels were quantified through quantitative reverse transcription polymerase chain reaction (RT-PCR). (**B**) Following CaMKKβ knockdown via RNA interference, HT29 cells were treated with 2 mM NaB for either 24 h2 h. Western blotting with densitometric quantification was subsequently performed: the 24 h group was used to assess LC3II levels, whereas the 2 h group was analyzed for AMPKα, ACC, p-CaMKKβ, p-AMPKα, and p-ACC expression. (**C**) Quantifying LC3 mRNA levels was done by quantitative RT-PCR. *P < 0.05, **P < 0.01, ***P < 0.001.

Initially, we investigated the impact of two distinct pairs of interference sequences targeting CaMKKβ. HT29 cells were subjected to a 24-hour treatment with either a non-targeting control sequence or the two specific interference sequences. Subsequently, the alterations in CaMKKβ mRNA expression levels were quantified using real-time fluorescence quantitative PCR. As depicted in Fig. 4A, both interference sequences, CaMKKβ siRNA-1 and CaMKKβ siRNA-2, resulted in a significant downregulation of CaMKKβ mRNA expression in HT29 cells, achieving interference efficiencies exceeding 70%. These findings validate the efficacy of the two interference sequences targeting CaMKKβ, indicating their suitability for further experimental investigations.

To systematically evaluate the impact of down-regulating CaMKKβ expression on autophagy in NaB-induced colorectal cancer cells, we established six experimental groups: the negative control group for CaMKKβ (NC), the positive control group with NaB treatment (NC combined with NaB treatment), CaMKKβ siRNA-1, CaMKKβ siRNA-1 combined with NaB treatment, CaMKKβ siRNA-2, and CaMKKβ siRNA-2 combined with NaB treatment. HT29 cells were transfected with three independent CaMKKβ-specific siRNA duplexes or scrambled control for 48 h, then treated with or without 2 mM NaB for the indicated durations prior to downstream analyses. The effects of CaMKKβ down-regulation on NaB-induced autophagy in colorectal cancer cells were evaluated using Western blotting and real-time quantitative PCR. The research results indicate that the down-regulation of CaMKKβ expression led to a significant reduction in the level of LC3 mRNA expression in colorectal cancer cells induced by NaB (refer to Fig. 4B). RNA interference-mediated suppression of CaMKKβ expression in colorectal cancer cells led to a significant decrease in CaMKKβ protein phosphorylation, comparable to the effect observed with STO-609, a known CaMKKβ inhibitor (see Fig. 4C). In order to provide additional validation regarding the impact of NaB inhibition on CaMKKβ and its subsequent signaling cascade, we assessed the phosphorylation status of AMPKα and ACC proteins, both of which serve as downstream targets of CaMKKβ. Following interference with colorectal cells, the specific sequences targeting CaMKKβ demonstrated a significant inhibition of NaB-induced p-AMPKα and p-ACC protein expression, as depicted in Fig. 4C. More importantly, the results depicted in Fig. 4C demonstrate a significant reduction in the level of LC3Ⅱ protein expression in colorectal cancer cells treated with NaB when CaMKKβ was down-regulated. These findings suggest that CaMKKβ signaling was involved in NaB-induced autophagy in colorectal cancer.

### Ca^2+^/CaMKKβ signaling was involved in NaB-induced autophagy in colorectal cancer

**Fig. 5:**
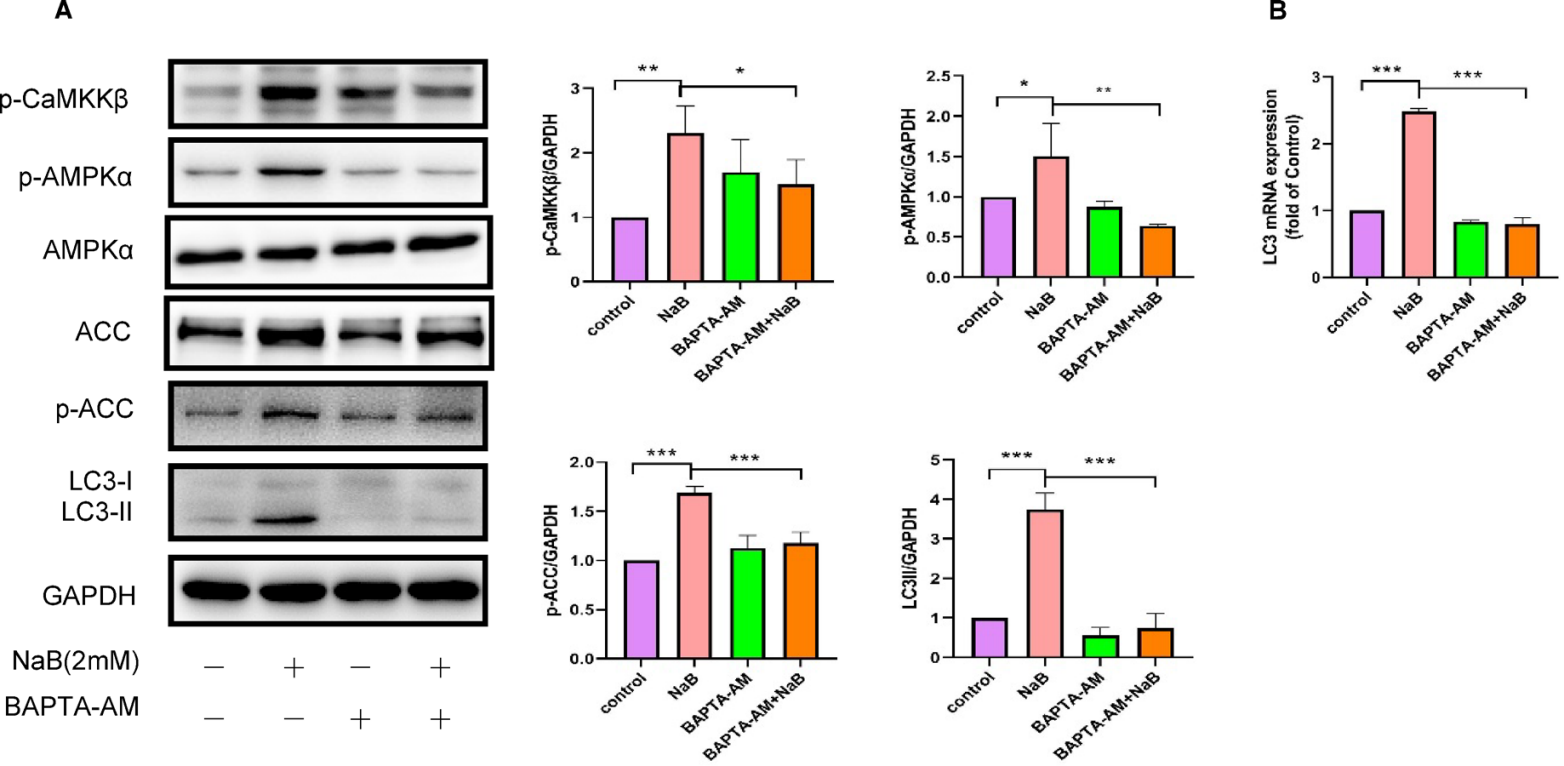
The induction of autophagy in colorectal cancer cells by NaB is impeded by the chelation of cytoplasmic calcium ions with BAPTA-AM. (**A**) colorectal cancer cells were pretreated with 5 µM BAPTA-AM for 30 min and subsequently exposed to 2 mM NaB for either 24 h–2 h. Western blotting followed by densitometric quantification was performed: the former condition was used to quantify LC3-II protein levels, whereas the latter was employed to evaluate the expression of AMPKα, ACC, p-CaMKKβ, p-AMPKα, and p-ACC. (**B**) By using quantitative RT-PCR, we quantified the mRNA levels of LC3. *P < 0.05, **P < 0.01, ***P < 0.001.

Research has highlighted the significant role of Ca^2+^ in regulating autophagy^16,26^. The increase in intracellular Ca^2+^ levels triggers the activation of various autophagy signaling kinases and proteasomes, ultimately leading to the induction of autophagy^27,28^. Furthermore, CaMKKβ, as a downstream effector in the classical Ca^2+^ signaling pathway, exhibits altered activity in response to fluctuations in Ca^2+^ concentration^26,29^. To determine whether Ca^2+^ signaling is required for NaB-induced autophagy in colorectal cancer cells, cells were pretreated with the intracellular Ca^2+^ chelator BAPTA-AM (10 µM) for 30 min prior to exposure to 2 mM NaB for the indicated durations. In order to elucidate the impact of chelating cytoplasmic calcium ions on CaMKKβ and downstream signaling molecules, the phosphorylation levels of CaMKKβ, AMPKα, and ACC proteins were assessed. The results indicated that pretreatment with BAPTA-AM effectively inhibited NaB-induced phosphorylation of CaMKKβ, AMPKα, and ACC proteins, as shown in Figs. 5A. Moreover, relative to the NaB treatment group, the expression level of the LC3II protein was markedly inhibited in the group receiving the combined BAPTA-AM and NaB treatment (refer to Fig. 5A). In a similar vein, the expression of LC3 mRNA was significantly reduced in the BAPTA-AM combined with NaB treatment group compared to the NaB treatment group (refer to Fig. 5B). These observations provide additional evidence that the Ca^2+^/CaMKKβ signaling pathway is implicated in NaB-induced autophagy in colorectal cancer.

## Discussion

**Fig. 6:**
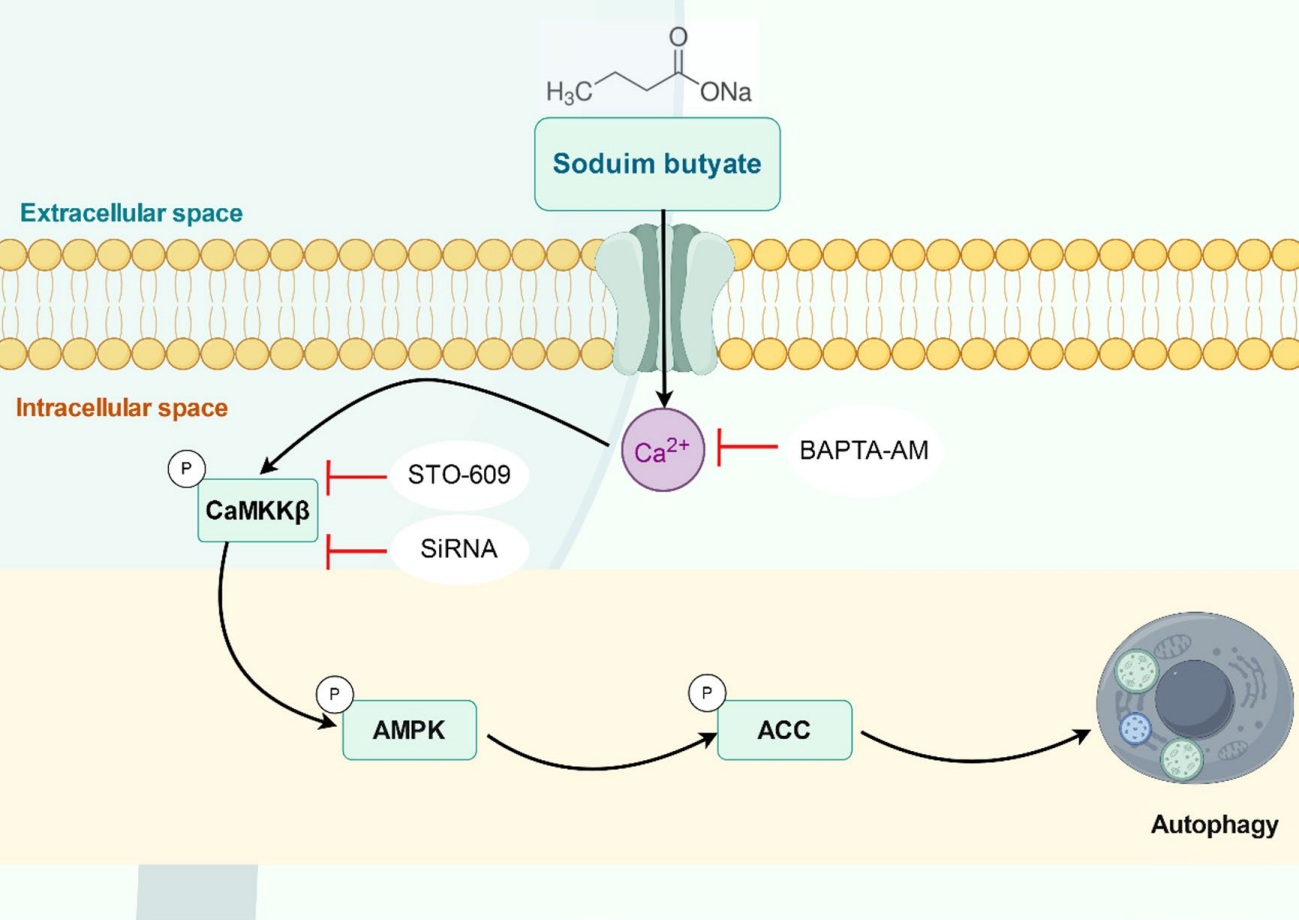
NaB induces autophagy in colorectal cancer cells via activation of the Ca²⁺/CaMKKβ signaling pathway. NaB induces autophagy in colorectal cancer cells by increasing cytosolic calcium ion concentration, thereby activating the CaMKKβ signaling pathway, which in turn activates AMPK and ACC signaling. *Note*:By Figdraw.

Dietary fiber is significantly associated with the nutritional prevention and treatment of colorectal cancer^30,31^. A growing body of research indicates that dietary fiber can mitigate the risk of developing colorectal cancer^32–34^. Numerous studies have validated the pivotal role of NaB in the anti-tumor effects attributed to dietary fiber^35,36^. Nonetheless, the precise anti-colorectal cancer effects and underlying mechanisms of NaB remain inadequately understood. Our research has demonstrated that NaB elevates cytosolic calcium ion concentration, thereby activating the CaMKKβ signaling pathway. This activation subsequently stimulates AMPK and ACC signaling, ultimately inducing autophagy in colorectal cancer cells. For further details, refer to Fig. 6.

The autophagic process involves the formation of autophagosomes, which are bilayer membranes encapsulating organelles, proteins, and other elements of the cell. After being fused with lysosomes, autophagosomes are able to degrade their contents to support the metabolic needs of the cell and to facilitate organelle renewal. The LC3 protein plays a crucial role in autophagy^37,38^, existing in two distinct forms within cells: LC3I and LC3II^39,40^. In autophagy, LC3I undergoes phospholipidation to form LC3II, leading to an increase in LC3II expression, which is localized to the autophagosome membrane^40^. Consequently, the heightened expression and translocation of LC3II are commonly regarded as indicators of autophagy. The findings of this research indicate that NaB can elevate the levels of LC3II protein in colorectal cancer cells, as well as facilitate the formation of autolysosomes. As a result of these findings, NaB might enhance autophagy in colorectal cancer cells.

CaMKKβ is a serine/threonine protein kinase that is intricately linked to Ca^2+^. Recent research indicates that the Ca^2+^/CaMKKβ/AMPK/mTOR signaling plays a crucial role in the regulation of autophagy^26^. Similarly, several studies have demonstrated that amino acid deprivation modulates autophagy through the Ca^2+^ level-dependent CaMKKβ-AMPK signaling, which inhibits mTORC1 and promotes autophagy^41^. Additionally, propofol has been found to inhibit autophagy in neuronal damage induced by oxygen-glucose deprivation through the Ca^2+^/CaMKKβ/AMPK/mTOR pathway^29^. The study demonstrated that STO-609, an inhibitor of CaMKKβ, attenuated the autophagy effect of colorectal cancer cells mediated by NaB and decreased the activity of CaMKKβ, AMPKα, and ACC. Similarly, silencing CaMKKβ expression through RNA interference also reduced the NaB-mediated autophagy effect and down-regulated the activity of CaMKKβ, AMPKα, and ACC. As a result, we have reason to believe that CaMKKβ signaling is critical to NaB-induced autophagy in colorectal cancer cells.

Traditionally, research in metabolic syndrome and obesity has primarily focused on Acetyl-CoA carboxylase (ACC). However, recent studies have revealed that ACC expression is increased in various cancers, and inhibiting ACC expression through ACC inhibitors or RNA interference can induce apoptosis in colorectal cancer cells^42^. ACC may play an important role in cancer cell survival, according to these findings. Additionally, research has shown that aspirin induces senescence via the SIRT1/AMPK/ACC signaling pathway, demonstrating its potential as an anti-colorectal cancer agent^43^. A growing body of evidence highlights ACC as a key regulator in cellular autophagy. For instance, Jie Gao and colleagues demonstrated that PRRX1 overexpression inhibits ACC1, thereby curtailing fatty-acid synthesis and the membrane lipid supply required for autophagosome biogenesis; consequently, LC3-II and Beclin-1 levels decline, autophagy is suppressed, and salivary adenoid cystic carcinoma invasion is accelerated^44^. Interestingly, another study has shown that in aging yeast, active Acc1 enhances de novo lipogenesis, providing lipids essential for autophagosome–vacuole fusion; conversely, Acc1 inhibition disrupts this lipid supply, thereby stalling autophagy and shortening lifespan^45^. The findings of this study demonstrated that the sequestration of intracellular calcium ion by BAPTA-AM significantly attenuated NaB-induced ACC protein phosphorylation in colorectal cancer cells. Additionally, the down-regulation of CaMKKβ expression using STO-609, an inhibitor of CaMKKβ, or RNA interference, led to a significant reduction in NaB-induced ACC protein phosphorylation in colorectal cancer cells. Despite this, we did not investigate the effects of ACC inhibitors or RNA interference on NaB-induced autophagy in our study. It is therefore warranted to investigate whether ACC could be involved in NaB-mediated autophagy of colorectal cancer cells.

In addition to regulating a wide range of physiological functions, Ca^2+^ also plays a crucial role in gene transcription, cell death, autophagy, and certain pathological functions^46–50^. In normal physiological conditions, intracellular calcium ion concentration ([Ca^2+^]_i_) levels are kept low, but during periods of stress, [Ca^2+^]_i_ levels rise rapidly. Currently, there is ongoing debate regarding the correlation between cytoplasmic Ca^2+^ levels and autophagy^50,51^. Studies have shown, however, that increased cytoplasmic Ca^2+^ concentrations may actually promote autophagy^26,29,52–54^. In studies, polyphenols like resveratrol and EGCG were shown to elevate [Ca^2+^]_i_ levels, resulting in activation of CaMKKβ, activation of AMPK, and induction of autophagy^27^. The current investigation illustrates that the sequestration of [Ca^2+^]_i_ through BAPTA-AM reduces the autophagic responses induced by NaB in colorectal cancer cells, leading to decreased levels of phosphorylated CaMKKβ, AMPKα, and ACC. Our findings suggest that NaB can trigger autophagy in colorectal cancer cells through the Ca^2+^/CaMKKβ pathway, although additional research is necessary to elucidate the precise mechanism involved.

Emerging evidence indicates that autophagy exerts a context-dependent, dual role in oncological therapy. On the one hand, cytoprotective autophagy can attenuate the efficacy of radiotherapy and chemotherapy by sustaining tumor cell survival^55,56^; on the other hand, excessive or sustained autophagy may precipitate autophagic cell death or sensitise tumor cells to apoptosis^57^. Pharmacological inhibition of autophagy with 3-methyladenine (3-MA) has been shown to potentiate hypoxia-induced apoptosis in human colorectal cancer cells^58^, underscoring autophagy modulation as a potential therapeutic strategy. Our previous studies have demonstrated that NaB markedly increases autophagic flux in colorectal cancer cells; genetic or pharmacological inhibition of autophagy subsequently triggers apoptosis^59^. Thus, the induced autophagy may function as a cytoprotective mechanism against NaB-mediated apoptotic cell death. In this study, we demonstrated that the inhibition of CaMKKβ expression by STO-609, the downregulation of CaMKKβ expression through RNA interference, and the suppression of intracellular calcium levels using BAPTA-AM significantly impeded NaB-induced autophagy in colorectal cancer cells. Furthermore, we observed a suppression in the activation of CaMKKβ, AMPKα, and ACC. These findings indicate that Ca^2+^, CaMKKβ, AMPKα, and ACC are critical molecular targets involved in NaB-induced autophagy within colorectal cancer cells. This suggests that modulating cytosolic calcium levels and the activity of CaMKKβ, AMPKα, and ACC can effectively achieve NaB-induced autophagy. In conclusion, autophagy modulators that target the Ca^2+^/CaMKKβ signaling pathway show potential as therapeutic agents for colorectal cancer. Nevertheless, their application requires careful timing and combination with other therapies to optimize therapeutic efficacy while minimizing potential adverse effects. Further research and clinical trials are essential to refine the use of these modulators in the treatment of colorectal cancer.

In conclusion, the findings of this study suggest that NaB promotes autophagy in colorectal cancer cells via the Ca^2+^/CaMKKβ axis, potentially establishing a strong theoretical basis for NaB to be considered as a primary treatment option for colorectal cancer in the future. However, this study has certain limitations. Although 2 mM sodium butyrate demonstrated significant biological activity at the cellular level, its translation into a safe, effective, and reproducible in vivo dose has not yet been rigorously validated. Future work should utilize murine xenograft models and/or chemically induced colorectal cancer models to systematically evaluate the antitumor efficacy of sodium butyrate in vivo and to clarify its underlying mechanisms.

## Supplementary Information

Below is the link to the electronic supplementary material.

Supplementary Material 1

## Data Availability

All data are provided in the manuscript.
