# Supplementary material for: Sodium butyrate-induced autophagy in colorectal cancer unveils the Ca²⁺/CaMKKβ signaling pathway as a potential therapeutic target
Source: Sci Rep. 2025 Dec 11;16:261. doi: 10.1038/s41598-025-29618-7 (PMC12770509; doi:10.1038/s41598-025-29618-7)
Supplement: Supplementary file 1 — Supplementary Material 1 [file 41598_2025_29618_MOESM1_ESM.pdf]

Supplementary Information Document

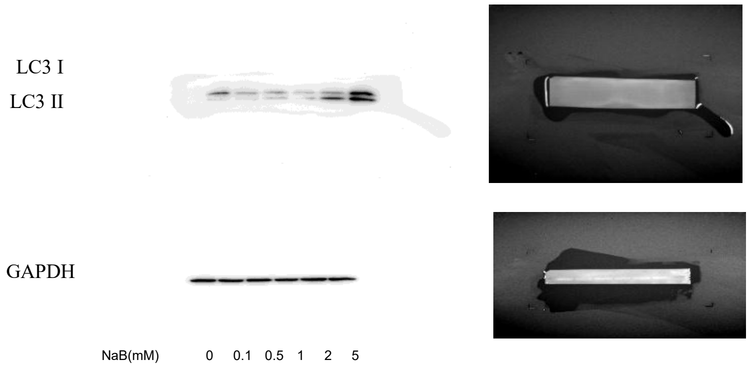

Figure 1 Impact of NaB on autophagy in colorectal cancer cells.

Note: The strip images and white light photographs used in Figure 1 are as shown above. We utilized the complete, full-length gels and blots without any additional cropping or alterations.

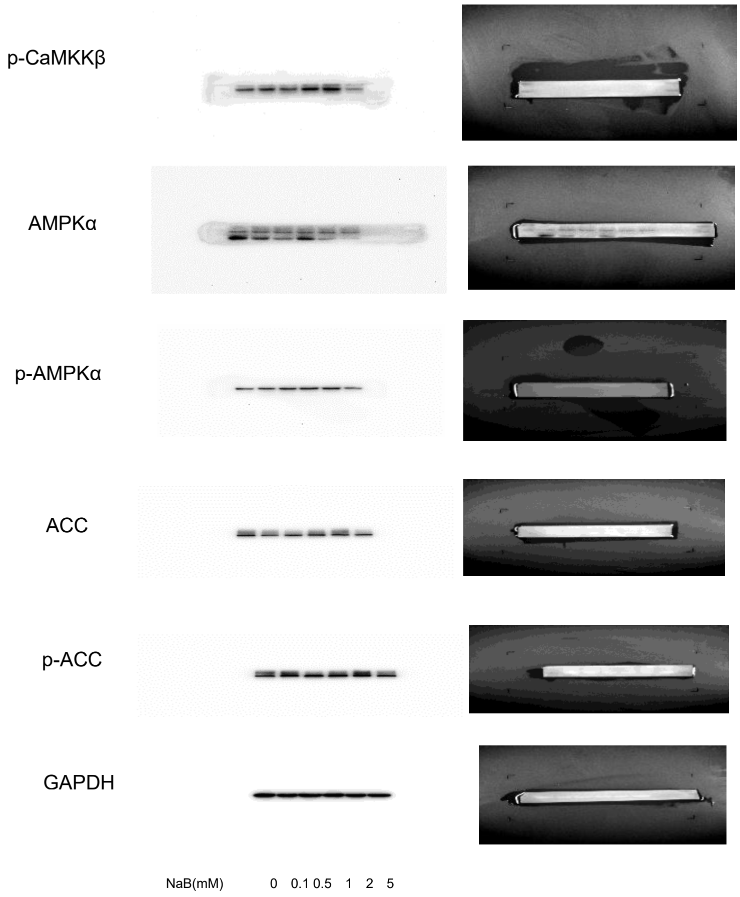

Figure 2 Activation of CaMKKβ, AMPK, and ACC by NaB in colorectal cancer cells.

Note: The strip images and white light photographs used in Figure 1 are as shown above. We utilized the complete, full-length gels and blots without any additional cropping or alterations.

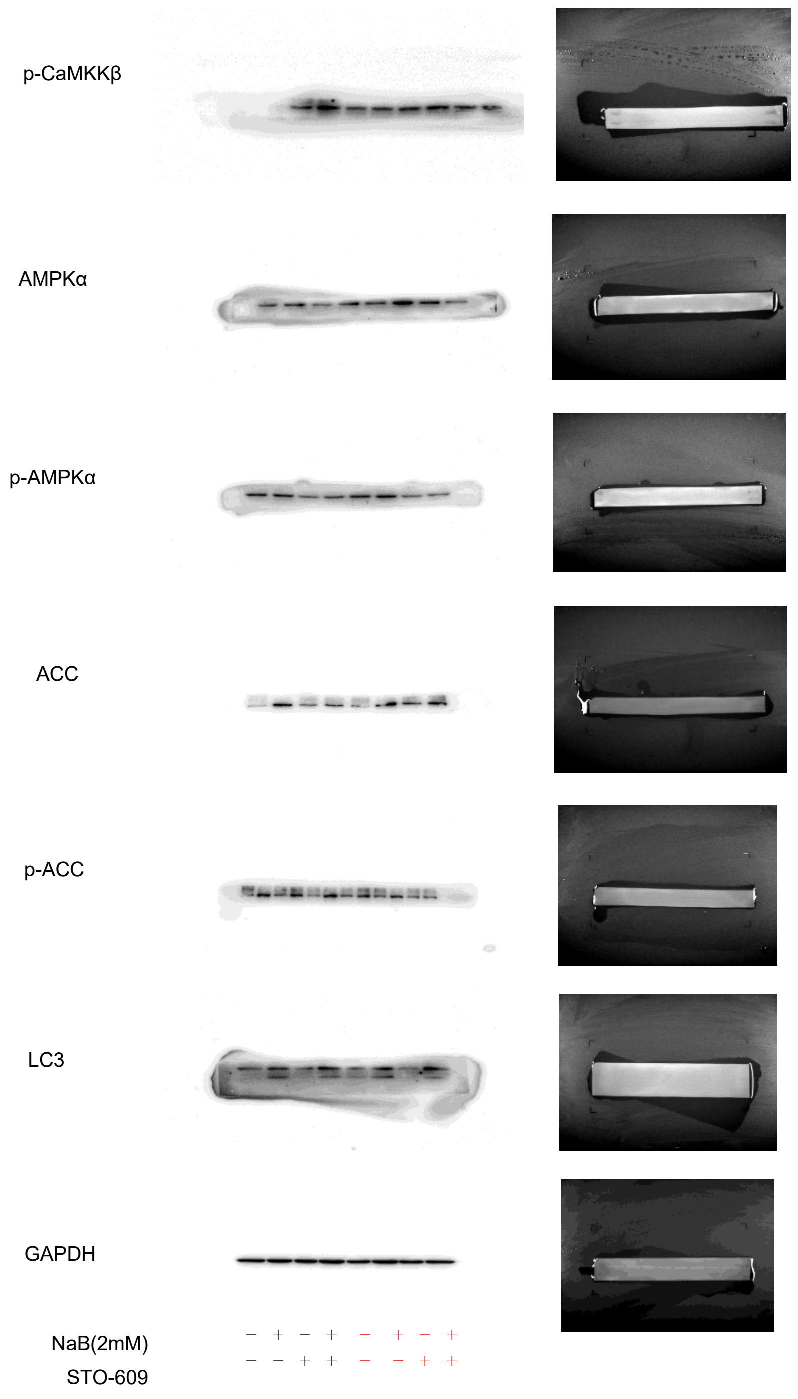

Figure 3 Inhibition of CaMKKβ activity attenuated NaB-induced autophagy in colorectal cancer cells

Note: The representative blot and bright-field images shown in Figure 3 are presented above. In the figure, results from two independent experiments are displayed (lanes 1–4 correspond to the first experiment, and lanes 5–8 to the second). One of thesexperimental results was selected as the

representative image included in the manuscript. Full-length, uncropped gels and blots were used without any additional trimming or splicing.

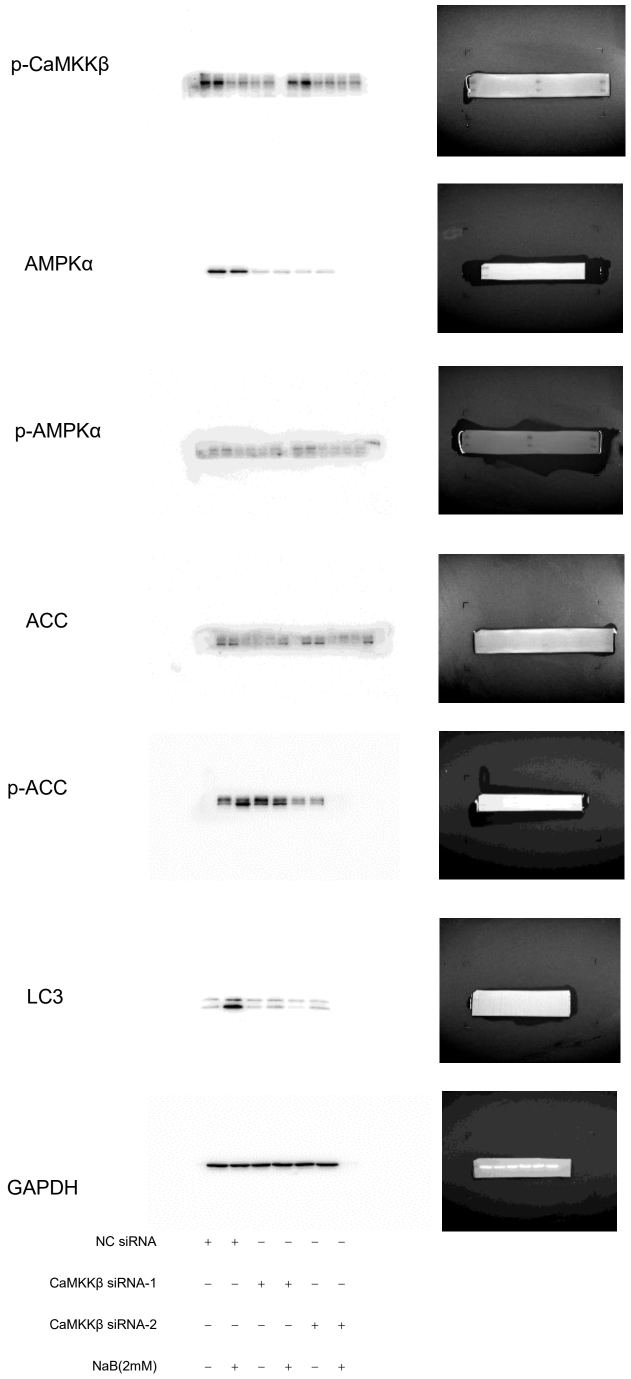

Figure 4 Reducing CaMKKβ expression via RNA interference decreased NaB-mediated autophagy in colorectal cancer cells

Note: The representative blot and bright-field images shown in Figure 4 are presented above. For the p-AMPKα and ACC blots, results from two independent experiments are displayed (lanes 1–6 correspond to the first experiment, lane 7 is the protein marker, and lanes 8–13 correspond to the

second experiment). One of these experimental results was selected as the representative image included in the manuscript. For all other protein blots, full-length, uncropped gels and blots were used without any additional trimming or splicing.

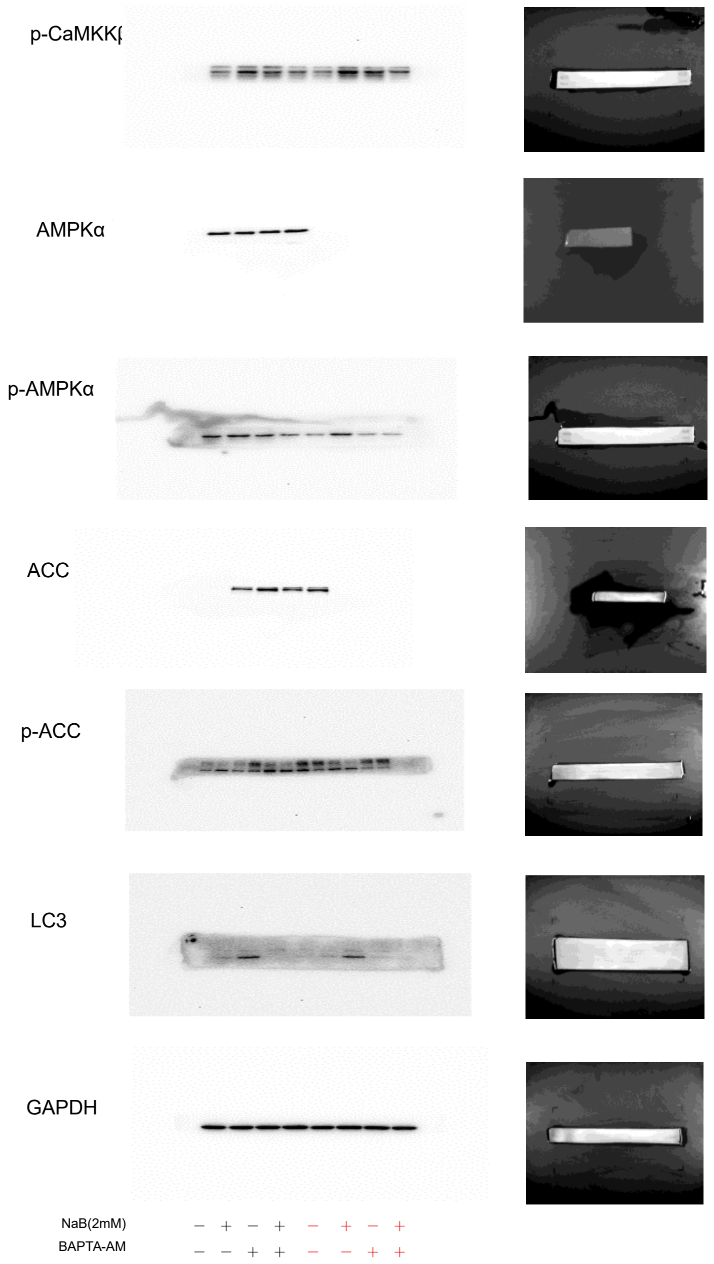

Figure 5 The induction of autophagy in colorectal cancer cells by NaB is impeded by the chelation of cytoplasmic calcium ions with BAPTA-AM

Note: The representative blot and bright-field images shown in Figure 5 are presented above. For the blots of p-CaMKKβ, p-AMPKα, p-ACC, LC3, and GAPDH, results from two independent experiments are displayed (lanes 1–4 correspond to the first experiment, and lanes 5–8 correspond to the second experiment). One of these experimental results was selected as the representative image included in the manuscript. For the ACC and AMPKα blots, full-length, uncropped gels and

blots were used without any additional trimming or splicing.
